# Second-generation extracellular matrix patch for epicardial infarct repair

**DOI:** 10.1186/s13019-023-02358-3

**Published:** 2023-09-01

**Authors:** Arjun Bhatt, Michael J. Bates, Constantin B. Marcu, Robert G. Matheny, Blase A. Carabello, Kanhua Yin, Walter Douglas Boyd

**Affiliations:** 1 Department of Cardiovascular Sciences, Brody School of Medicine, East Carolina University, 115 Heart Drive, Greenville, NC 27834 USA; 2 CorMatrix Cardiovascular Inc, Roswell, GA USA

**Keywords:** Myocardial infarction, Ischemic heart disease, Extracellular matrix patch, Myocardial scar

## Abstract

Current myocardial infarction treatments focus on improving hemodynamics rather than addressing the problem of lost myocardium impairing left ventricular function. Epicardial infarct repair with a bioactive patch placed on the ischemic area is an emerging approach to promote endogenous myocardial repair. We report the use of a second-generation CorMatrix-extracellular matrix (ECM) patch as an adjunct to surgical revascularization in treating a young patient with diffuse, multivessel coronary artery disease unamenable to PCI and a large anterior myocardial infarction. The progressive myocardial scar shrinkage and increase in left ventricular ejection fraction from 10 to 51% are generally not observed with surgical revascularization therapy alone, suggesting this new patch has adjunctive potential to current revascularization therapy.

**Supplementary Information:**

The online version contains supplementary material available at 10.1186/s13019-023-02358-3.

## Introduction

Coronary revascularization is the primary therapeutic approach to restore perfusion and the standard care for acute myocardial infarction (MI). However, for post-MI patients with a large dyskinetic myocardial scar and significantly decreased left ventricular ejection fraction (LVEF), additional therapy is needed to further rescue the myocardium. Although more preclinical and clinical studies are still needed, epicardial infarct repair is considered an emerging technique where functional biomaterials are applied over the epicardium in the infarcted area to modulate pathways toward myocardial regeneration. This case report describes the in-human use of a second-generation CorMatrix-extracellular matrix (ECM) patch [1] in a young patient who suffered a large anterior MI resulting in severe left ventricular dysfunction.

## Case report

A previously well 29-year-old male with a PMH of morbid obesity and tobacco/marijuana abuse experienced sudden chest pain and persistent hypoxemia after exercising at home. CPR was delivered by family for approximately 30 min while awaiting EMS arrival. During EMS evaluation and transport, he had three cardiac arrests over 45 min, each with subsequent return of normal rhythm and circulation after cardiopulmonary resuscitation. He was intubated at an outside hospital, and after 3.5 h, transferred to our center, arriving six hours after symptom onset. An electrocardiogram confirmed anterior ST-segment elevation MI. Transthoracic echocardiography revealed apical, periapical, and anterior wall akinesis, global hypokinesis, and LVEF of 10%. Emergent coronary angiography demonstrated 100% mid-left anterior descending (LAD), 60% left circumflex artery, and 100% right coronary artery occlusions. No collateral perfusion was evident. Urgent mid-LAD angioplasty was performed, but due to ongoing hypotension and persistent hypoxia, the patient required support with veno-arterial extracorporeal membrane oxygenation (ECMO). Further treatment options were discussed with the family including the risks and benefits of continued medical therapy versus surgical revascularization as well as comfort care options. Additional options including the use of ventricular assist devices were discussed and after the patient had been on ECMO for > 24 h the family decide on surgical revascularization with potential device implantation.

**Fig. 1:**
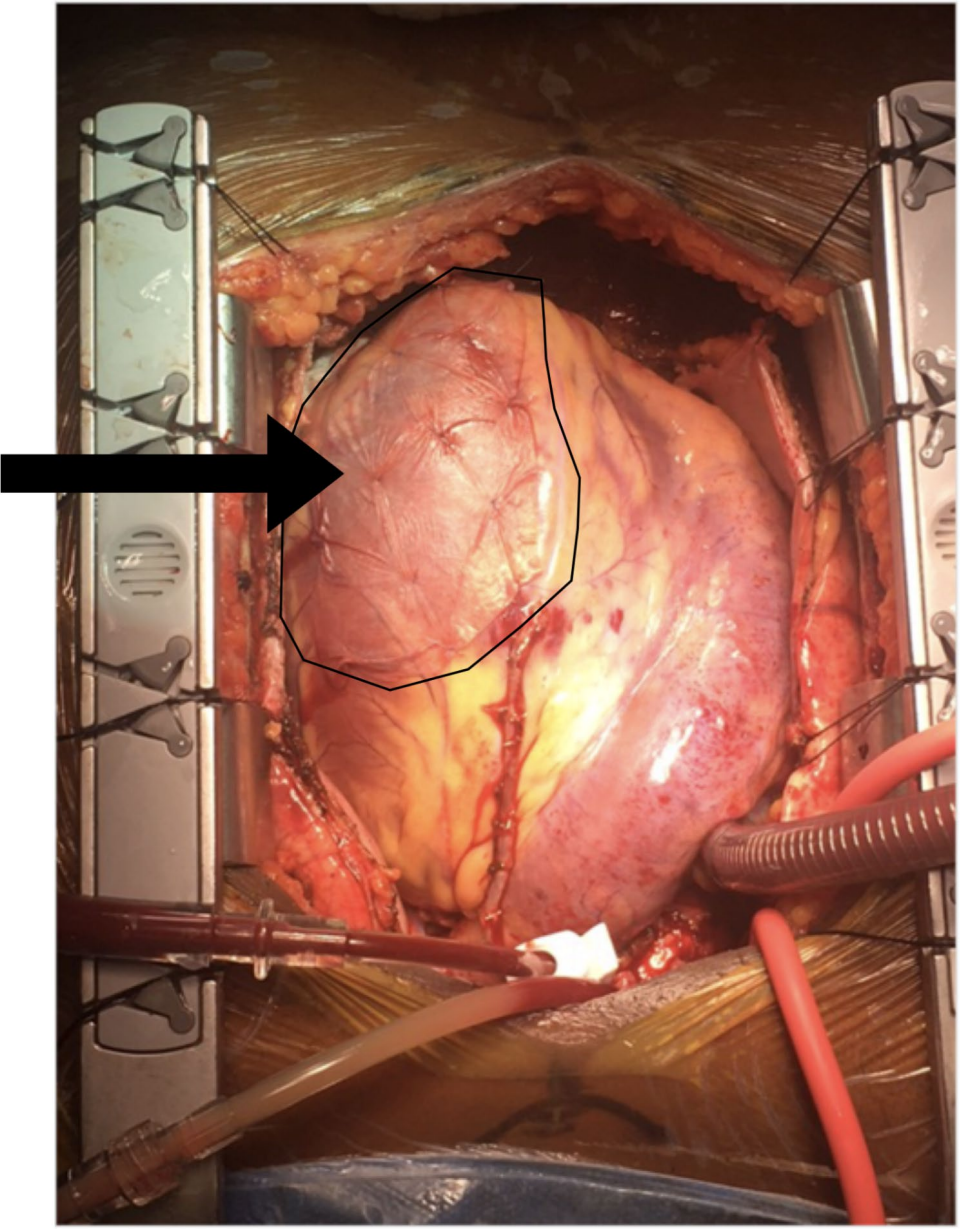
Intraoperative image showing the morphological fit of a second-generation CorMatrix-ECM patch applied to the infarcted region of the left ventricle

Total arterial coronary artery bypass was performed four days after admission, with the left internal mammary artery placed to the mid-LAD, right internal mammary artery to the posterior descending artery, and left radial artery to the first diagonal artery. Intraoperatively, an infarcted and nonfunctional anterior wall corresponding to the LAD perfusion territory was noted. A second-generation ECM bioscaffold patch, CorMatrix Cor™ PATCH (CorMatrix Cardiovascular, Inc., Roswell, GA), was epicardially applied to the infarcted ventricular territory using interrupted 5 − 0 Prolene (Fig. 1). Later, lateral and inferior left ventricular recovery allowed for weaning of ECMO.

The patient was extubated the following day. His postoperative course was complicated by diffuse anoxic brain injury resulting in a slow recovery of neurological deficits and prolonged hospital stay. On postoperative day (POD) 12, a late gadolinium enhancement cardiac magnetic resonance (LGE CMR) showed subendocardial LGE with variable extent from 50% in the mid-anteroseptal to 75% in the distal septal, distal anterior, and apical cap, suggesting a recent infarction with edema and myocardial fibrosis. This CMR reported an LVEF of 31%. He was discharged to a rehabilitation facility on POD 29 and home on POD 38.

**Fig. 2:**
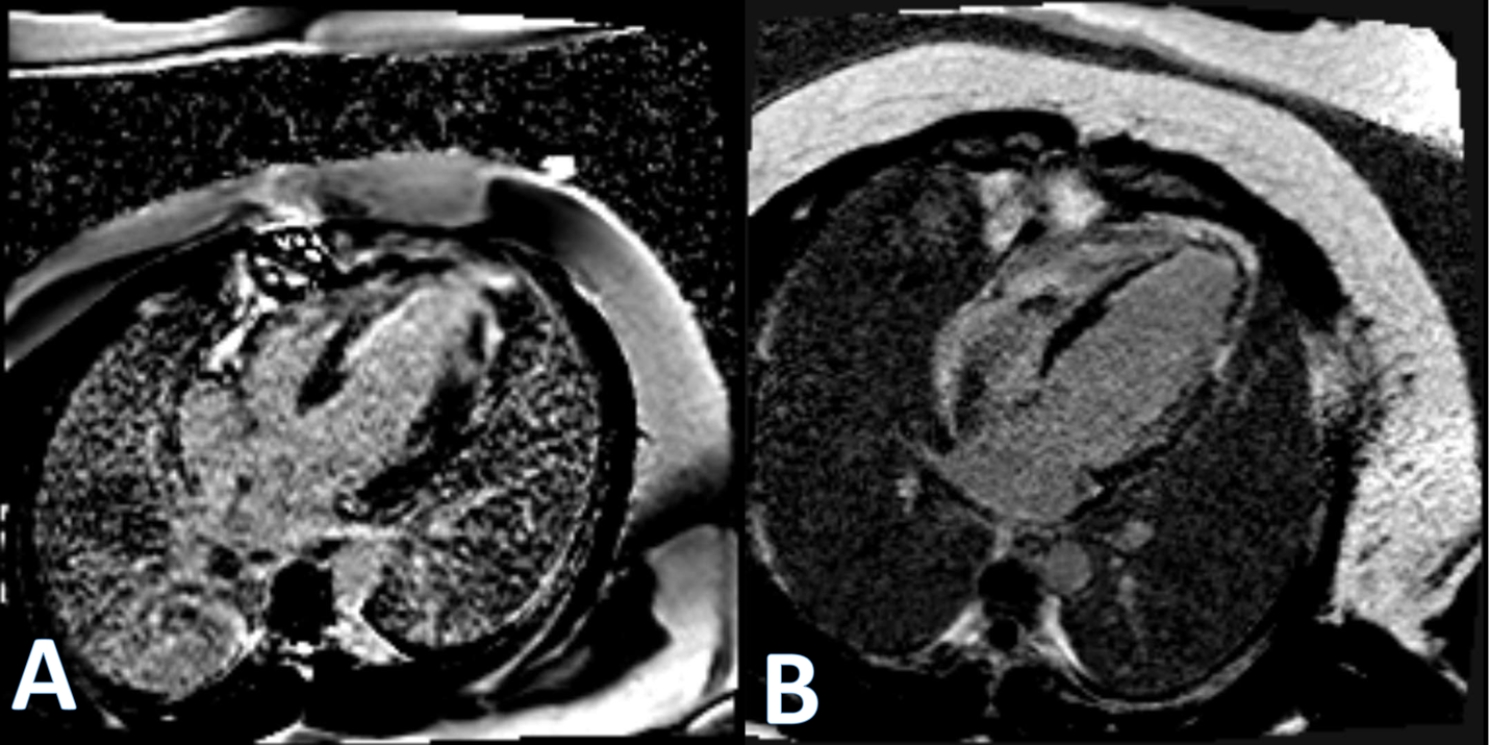
LGE CMR shows clear improvements in the extent of scar tissue between POD 12 (**A**) and POD 426 (**B**)

This patient was followed by a series of LGE CMR performed at five, 10, and 14 months after surgery; LVEF increased linearly with 39% in the fifth month, 45% in the 10th month, and 51% in the 14th month; cine comparing four-chamber views between POD 12 and 14th months is provided (Video S1). The myocardial fibrosis and scar steadily improved, evidenced by the decrease in the LGE extent (Fig. 2). In the most recent office visit (POD 732), the patient was doing well, tolerating daily 20 min walks without chest pain or dyspnea but experiencing dyspnea with more vigorous exercise. While the patient’s cardiovascular symptoms are consistent with New York Heart Association Class II and has no complaints regarding his exercise tolerance, however he does experience sequelae of his anoxic brain injury, specifically bilateral foot drop and some persistent cognitive impairment.

## Discussion

This young patient presenting with cardiogenic shock sustained a remarkable improvement in LVEF post-operatively. The obvious issue is whether this improvement resulted from revascularization restoring function to hibernating myocardium, medical therapy, or cardiac regeneration catalyzed by bioactive acellular ECM patch placement. Reperfusion improvement in ventricular function has been documented over decades and is not novel. However, the scar that develops post-MI is not contractile and seldom becomes so after revascularization; restoration of function and decreasing scar burden has been a goal of post-MI therapy for many years.

Two major pieces of evidence support that in this case the functionalized matrix contributed to the clinical and MRI improvements seen. First, the natural history of MI is well-defined. Necrosis begins shortly after coronary occlusion; by three hours of total occlusion, most of the damage is done [2]. In this case, six hours elapsed between symptoms and reperfusion with no evidence of collateral circulation to mitigate damage. Thus, the natural history of MI indicates that a large infarction would have ensued possibly with aneurysm formation. Second, a series of postoperative LGE CMR, an in vivo gold standard for separating scar from viable myocardium, confirmed that a large scar was present; this scar regressed over time concomitant with LVEF improvement.

In this case, applying a second-generation CorMatrix-ECM patch to the infarcted area helped preserve and restore cardiac function by two possible mechanisms. First, the mechanical constraint has been demonstrated to improve ventricular performance after MI [3]. This can be achieved with many patch materials, including synthetics, but these patches often cause secondary problems due to their inherent inflammatory nature. However, the ECM patch from porcine small intestine submucosa demonstrably prevents inflammation and scarring [1]. The second mechanism is via the delivery of paracrine factors bound within the matrix that are released into the epicardium. These factors, including various growth factors and matrix-bound vesicles, are transported to the subepicardial and mobilize resident repair cells to enhance proliferation and produce microenvironments conducive to angiogenesis and tissue remodeling [4]. Rat coronary artery ligation models demonstrate that epicardial implantation of the CorMatrix-ECM patch is associated with an increase in basic fibroblast growth factor expression, which can contribute to the improvement of LVEF [5]. Clinical studies have also been reported using an ECM patch for mitral valve repair and treatment of ventricular septal defects [6, 7]. The second-generation CorMatrix-ECM patch used in our case was developed to further reduce xenogeneic nucleic material, cell wall fragments, and lipids to reduce antigenicity without significant increases in surgical complexity relative to revascularization alone.

Additionally, LV mechanical support after MI, such as intracorporeal support and off-loading (Impella), has been shown to decrease infarct size. It is compelling that therapies may be adjunctive in reducing the sequelae of MI by utilizing mechanical off-loading and the addition of a biologically active patch to decrease inflammation and activate endogenous regeneration.

In conclusion, this case report describes the successful implantation of a second-generation CorMatrix-ECM patch as adjuvant therapy to restore cardiac function in an anterior MI patient who was complicated with cardiogenic shock and left ventricular dysfunction. Serial LGE CMRs demonstrated progressive favorable left ventricular remodeling and more than expected myocardial scar shrinkage with the possible restoration of myocardial tissue. Our data support a prospective effort to analyze the possible clinical benefit of a functionalized patch in the presence of both acute and chronic myocardial infarction. More clinical observational studies or large-scale clinical trials are warranted to further confirm or disapprove these effects.

### Electronic supplementary material

Below is the link to the electronic supplementary material.

Supplementary Material 1

## Data Availability

The raw data are available on reasonable request due to privacy or ethical restrictions.
